# 

**Published:** 1842-06

**Authors:** 


					1
Volume II.
JUNE, 1842.
no. y
THE
AMERICAN
JijptNAL AND LIBRARY
llaPl^r
or
IB exit a I Q txtntt.
PUBLISHED UNDER THE AUSPICES OF THE
afttterCcan Sb'ocfetg of liental Sttrseons,
EDITED BY
CHAPIN A. HARRIS, M. D., Baliimoke,
AND
SOLYMAN BROWN, M.D., New Yobi.
NEW YORK:?GEORGE ADLARD,
BALTIMORE :-ARMSTRONG 8c BERRY,
LONDONSAMUElj fflGHLEY;
WOODS AKD CRANE, PR8., SALT*
8500 per annum, payable in advance.
This number contains 9 sheets.
? 09 A H IT B K G, V

				

